# Qatar's National Mental Health Survey—The World Mental Health Qatar: Sampling design, instrument adaptation, quality control, and fielding procedures

**DOI:** 10.1002/mpr.2010

**Published:** 2024-05-10

**Authors:** Salma M. Khaled, Iman Amro, Lina Bader, John Lee Holmes, Kien Le Trung, Abdoulaye Diop

**Affiliations:** ^1^ Department of Population Medicine College of Medicine Qatar University Doha Qatar; ^2^ Social and Economic Survey Research Institute Qatar University Doha Qatar

**Keywords:** CATI—computer assisted telephone interviewing, CIDI—composite international diagnostic interview, COVID‐19 pandemic, Qatar, world mental health survey initiative

## Abstract

**Objectives:**

The World Mental Health Qatar (WMHQ) study, the first national general population mental health survey in Qatar, was conducted as part of the World Health Organization (WHO) World Mental Health (WMH) Survey Initiative. It was one of the few WMH survey conducted during the COVID‐19 pandemic. This paper presents the methodological advances and challenges encountered while conducting the survey by telephone during the pandemic.

**Methods:**

Disproportionate stratified sampling using a national‐level cellular telephone frame selected a representative sample of Arabic‐speaking adults. Participants were initially contacted via Short Message Service text, followed by telephone interviews. WMH training materials supported a comprehensive training program, and data quality was ensured through a quality control indicator system and extensive monitoring.

**Results:**

Over 234 days, 5195 interviews in Arabic were completed, averaging 77 min each. In line with Qatar's population, the majority of participants were non‐Qatari residents living in Qatar (72.2%).

**Conclusions:**

A distributed remote Computer Assisted Telephone Interviewing system facilitated centralized quality monitoring and data security. However, the pandemic intensified challenges such as remote management of interviewer productivity, low response rates, and rising survey costs. The findings will inform Qatar's mental health policymakers, and the strategies used to address these challenges offer valuable insights for researchers worldwide.

## 1 INTRODUCTION

The World Mental Health (WMH) Survey Initiative, developed by the World Health Organization (WHO), has conducted and analyzed epidemiological survey data on the prevalence and correlates of mental disorders and their treatment in more than 30 countries to date (Alonso et al., 2013; Kessler et al., 2008; Scott et al., 2018). All WMH surveys use the same survey instrument, the Composite International Diagnostic Interview (CIDI), a fully structured diagnostic interview that generates diagnoses according to the definitions and criteria of the WHO International Classification of Diseases and the American Psychiatric Association Diagnostic and Statistical Manual of Mental Disorders (DSM). The WMH surveys used consistent interviewer training programs, materials, and quality control (QC) monitoring procedures to ensure comparability across countries.

We planned to use similar methods for conducting the World Mental Health Qatar (WMHQ)—the first national epidemiological survey of mental health in Qatar's general population. Qatar joined the WMH consortium in January of 2019. However, due to the COVID‐19 pandemic, the original study methodology underwent extensive revisions, most notably replacing household‐based face‐to‐face interviews with phone interviews. This necessitated repeating some of the piloting and instrumentation phases that had already taken place for the face‐to‐face survey fielding prior to launching the telephone survey. For example, the pre‐pandemic face‐to‐face pilot conducted between January and February of 2020 was repeated as a phone pilot between October 2020 and January 2021.

An overview of the WMHQ study was presented in a prior paper in this issue (Khaled, Al‐Abdulla, et al., 2024). Briefly, we aimed to establish a national capacity to conduct research in population mental health and track changes across time by establishing robust baseline estimates of the lifetime and 12 months prevalence of common psychiatric disorders. Nationally representative data on prevalence, predictors, and treatment contact for mental disorders in Qatar are not available to date. Having this data will further inform the direction and focus of community‐based mental health services in the country. A paper discussing issues related to survey mode emerging from the two study pilots conducted prior to and during the COVID‐19 pandemic was also presented earlier in this issue (Khaled, Amro, et al., 2024). In the current paper, we aim to describe the revised methodology for the full‐scale production of the WMHQ survey, including sample design, instrument features, and fielding procedures.

## 2 METHODS

### 2.1 Sample design

The target population for the study comprised Arab adults 18 years of age or older residing in Qatar during the survey reference period (January 2019–January 2022). Respondents were surveyed using a Computer Assisted Telephone Interviewing (CATI) system along with other assisting technology.

Working with local cell phone providers in Qatar, the Social and Economic Survey Research Institute (SESRI) at Qatar University developed a cell phone frame suitable for the survey. As the vast majority (98%) of adults in Qatar have at least one cell phone, the frame was expected to provide excellent coverage for this target population (SESRI Omnibus, 2019). The frame included auxiliary information that was used to improve the efficiency of the sampling process.

**FIGURE 1:**
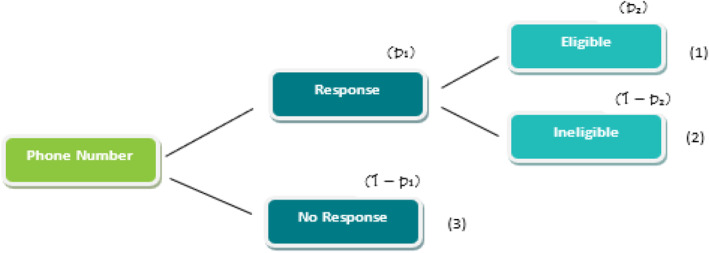
Dialing outcomes–phone number states and probabilities. A phone number could be in one of the following three states: (1) a phone number with an Arab adult eligible for the survey; (2) an ineligible person; or (3) no‐response. *p*_1_ is the probability of a response; (1‐*p*_1_) is the probability of non‐response; *p*_2_ is the probability of being eligible conditional on response; similarly (1‐*p*_2_) is the conditional probability of being eligible and *p*_1_ (1–p_2_) is the probability for being ineligible.

In the survey component of the study, the disposition from dialing a phone number can be described in two stages (Figure 1). First, a response or no response (e.g., non‐working or disconnected numbers, immediate hang‐up, or refusal) was obtained from the dialing procedure. Then, in the second stage, a phone number with a response was identified as eligible (Arab adults) or ineligible (e.g., less than 18 years old, non‐Arab adults). “Arab status” was determined based on two criteria: nationality and language. Arabs who did not speak Arabic were not included.

As shown in Figure 1, there were three possible states for a phone number. A phone number could be (1) associated with an Arab adult eligible for the survey, (2) associated with an ineligible person, or (3) a non‐response. Since we did not know the state of a phone number prior to dialing, the sampling process was conducted while the state of each phone number was unknown. Accordingly, a simple random sample would be quite inefficient and associated with a high survey cost, as a large number of sampled phone numbers would end up in ineligible or no‐response states.

Based on the sampling literature for rare population surveys (Chen & Kalton, 2015; Kim et al., 2014; Sanchez et al., 2009; Waksberg et al., 1997), SESRI developed a sampling process to address this issue. First, we used data from previous phone surveys where the states of the phone numbers had been identified after dialing. This data was applied to a two‐stage (or nested) logistic regression. In the first stage regression, the dependent variable was a response or no response, and in the second stage regression, the dependent variable was eligible or ineligible. For both stage‐regressions, the independent variables were derived from auxiliary information in the frame. Following these regressions, the probabilities *p*_1_ and *p*_2_ in Figure 1 can be calculated as follows:

$$p_{i}=\frac{e^{x_{i}\beta_{i}}}{1+e^{x_{i}\beta_{i}}}$$

where *i* is 1 or 2, *x*_*i*_ is a vector of independent variables, and *β*_*i*_ is a vector of estimated coefficients from the nested logistic regressions.

The probability for each state will be the product of these two probabilities; that is, *p*_1_ *p*_2_ for eligible, *p*_1_(1 − *p*_2_) for ineligible, and 1 − *p*_1_ for non‐response. Since the independent variables were derived from the auxiliary information in the frame, these probabilities can be extrapolated to all phone numbers in the frame. In other words, for every phone number in the frame, we can calculate its probability of belonging to state 1 (eligible), 2 (ineligible), or 3 (non‐response).

Next, using these probabilities, we divided the frame into three strata in descending order of probability of selection. The first stratum included phone numbers that were most likely to be eligible, while the last stratum consisted of phone numbers least likely to be eligible, most likely including ineligibles and no‐responses.

Finally, we constructed a disproportionate stratified sample from these strata. The disproportionate allocation was important to achieve efficiency, applying a higher sampling fraction to the stratum with a higher probability of eligibility. With this allocation, we were able to reduce survey costs as the sample was more likely to contain eligible phone numbers.

In fact, we achieved an optimal allocation of the sample into these strata by solving the optimization problem in which the objective function was the variance of an estimated mean, $Var\left(\bar{Y}\right)$, with the survey cost as the constraint. The optimal sampling fraction derived from this optimization was:

$$f_{h}\propto \sqrt{\frac{P_{h}}{P_{h}\left(c-1\right)+1}}$$

where *P*_*h*_ is the proportion of the eligible phone numbers in stratum *h*, and *c* is the ratio of the data collection cost for eligible phone numbers to that for ineligible phone numbers. Further details of this optimization problem and its solution can be found elsewhere (Barron et al., 2015; Chen & Kalton, 2015; Kalton, 2009).

### 2.2 Survey instrument

When the WHO declared COVID‐19 a global pandemic, the WMHQ study team began revising the original study methodology. This involved conducting phone interviews using CATI technology rather than household‐based face‐to‐face interviews using Computer Assisted Personal Interviewing (CAPI) technology. The CIDI version 3.3 was used to assess the burden of mental illness during these interviews, based on the fifth edition of the DSM (DSM‐5; American Psychiatric Association, 2013).

The instrument translation and adaptation to Arabic began prior to the face‐to‐face pilot and underwent testing and modification during and after this first study pilot. This iterative process has been described in detail in another publication (Khaled et al., 2021). The shortening of the instrument for its adaptation to the phone pilot was described in an earlier manuscript in this issue (Khaled, Amro, et al., 2024).

#### 2.2.1 Instrument sections by interview type (long versus short)

In order to manage the length of the interview and minimize survey break‐off, we customized the survey so that only those who met the minimum criteria of lifetime mental illness were required to complete the long form of the interview. This was achieved using an algorithm where we divided all respondents into one of three groups at the end of the short interview for the WMHQ instrument: (1) those with “threshold” disorders, (2) those with “sub‐threshold” disorders, and (3) all others. The algorithm selected 100% of the people in the first group, 50% in the second group, and 25% in the third group for the long form. We applied weights to the final dataset to adjust for the under‐sampling of respondents in the second and third groups, ensuring that the weighted prevalence of disorders in the final sample had the same expected value as in the total original sample.

As shown in Table 1, the content of the long interview was the same as that of the short interview, with the exception of the following additional sections: obsession and compulsions (obsessive compulsive disorder), stressful experiences (post‐traumatic stress disorder), and treatment.

**TABLE 1:** Content summary of the final version of the WMHQ instrument by interview type.

Sections	Contents	Details
Socio‐demographic	Background	Gender, age, education level, physical health, contact details, marital status, children and other sociodemographic, employment status, financial status
	Respondent contacts	
	Health	
	Employment	
	Finance	
COVID‐19^b^	Exposure (self, family, coworkers)	Experiences with lockdown and restrictions' impact on mental health.
	Mental and physical impact	
		Mental health status and related symptoms during COVID‐19
	Lockdown measures	
Mood disorders	Depression	Major depression and mania, “symptoms and experiences”
	High mood	
Anxiety disorders	Worry and anxiety	General anxiety disorder symptoms, panic attacks, obsessions, and compulsions
	Panic attacks	
	Obsession and compulsions^a^	
Other sections	Unusual experiences	Different experiences throughout childhood and other unusual, stressful experiences, including hallucinations, delusions, psychosis, emotional difficulties, peer pressure, marriage problems, other stressful events
	Childhood experiences	
	Stressful experiences^a^	
Treatment section	Treatment^a^	Use of treatment for one or more mental disorders and type of treatment received
Non‐CIDI^c^ sections	Personal relationships	Specific personal experiences related to social relations and reactions to social events, assessing perceptions of possession of certain schizotypal personality traits
	Social interactions	
	Perceptual attitudes	

^a^ Sections included in the long interview only.

^b^ COVID‐19: Coronavirus disease of 2019.

^c^ Non‐CIDI sections: were not part of the CIDI instrument and were added based on researchers interest.

#### 2.2.2 Instrument programming and testing

The WMHQ survey instrument was implemented in the Blaise computer‐assisted survey interviewing system and processing tool for the Windows operating system (https://blaise.com/). The information technology (IT) team divided the programming of the questionnaire into two parts. The first part consisted of adding the final Arabic text approved by the translation team. The second part focused on debugging the survey to ensure that the questions, skip logic, and checkpoints were correct. Finally, test executions for each survey section were written to assist the research team in completing the survey. The survey underwent modifications during the programming process, which included adding, deleting, and altering questions and checkpoints.

The IT team received the requested changes from the research team via email and subsequently applied them in Blaise. The changes were then reviewed on the training version of the survey by the IT team to debug any issues with the programming script. Following this, the research team debugged the survey, approved the changes, or provided feedback to the IT team, repeating the process as necessary. Upon final approval, the survey was deployed on tablets for survey production.

Key IT technologies used in the course of the WMHQ study are summarized in Table 2.

**TABLE 2:** Key IT technologies in the WMHQ survey.

Blaise	CATI management system	Microsoft power BI
Blaise is computer‐assisted interviewing software. It is used to program a survey and deliver cases to agents using its CATI^a^ components.	A system with a scheduler that prioritizes the cases that need to be called at the right time. Also a dashboard to manage cases, appointments, user/account management, case delivery and error logs, and summarized reports.	Microsoft power BI^b^ is a reporting system used to visually present data to the study research team.
CATI time tracker	**Payroll system**	**Microsoft planner**
An application used to manage and track agent activities (calling and other administrative activities) and work shifts.	A payroll system was used to track agent profile details, survey history, payment history, and to calculate and process payments.	Microsoft planner is a task management system used to assign flag investigation tasks to quality control team members. It was used to track the status of the flags and document actions taken.
Cisco	**Microsoft SQL server**	**Team city**
Cisco is an IP telephony system integrated with blaise to prevent manual dialing of phone numbers. This system includes cisco IP^c^ communicator, a soft‐phone application used to initiate phone calls from agent workstations.	Microsoft SQL^d^ server, a relational database management system, was used for data storage and processing (ETL^e^).	Team city is a build management and continuous integration tool.
Power app	**Python**	**Blazor**
Power apps is a suite of apps and services used to develop the CATI^a^ time tracker app.	Python is a programming language used with data science libraries as a tool for data processing.	Blazor/C#^f^ is a web‐based framework used for developing the payroll app.
TeamViewer		
TeamViewer is a support software that allows IT^g^ support to remotely access and control agents' workstations.		

^a^ CATI: Computer Assisted Telephone Interviewing.

^b^ BI: Business Intelligence.

^c^ IP: Internet Protocol.

^d^ SQL: Structured Query Language.

^e^ ETL: Extract, Transform, Load.

^f^ C#: C‐Sharp, is a programming language developed by Microsoft.

^g^ IT: Information Technology.

#### 2.2.3 Interviewers' recruitment and training

Interviewers were selected based on their prior call center experience, reading, pronunciation, and persuasion skills, as well as their availability to work 32 h per week. Additionally, their personality traits, ethical behavior, and basic IT skills were evaluated. While a background degree in psychology was favored, it was not a requirement.

The training process involved three teams: the research team, the CATI lab team, and the IT team. The training sessions spanned five full days, supplemented by as‐needed online refresher sessions. Interviewer training began with an introduction to SESRI's phone lab, general interviewing techniques, and basic CATI interviewing procedures. The training team explained the questionnaire instrument, emphasizing informed consent and confidentiality. Role‐play and round‐robin style training methods were used to practice the pre‐prepared scripts for different case scenarios, including both short and long interview cases. These hands‐on sessions provided examples of key interviewing techniques, such as professional question reading and accurate entry of respondent responses and notes in accordance with the interview protocol and guidelines for the study.

On the last day of the training, mock interviews were conducted to select the most qualified interviewers for the study. Throughout the fielding of the study, the research team continuously maintained high survey data quality and monitored interviewers' behavior and performance. These field‐period strategies are described in detail under the QC section.

#### 2.2.4 Ethical consideration

Qatar University's ethics committee (QU‐IRB 1219‐EA/20) approved the survey component of the WMHQ study. The survey's procedures and aims were explained verbally to the participants. Before starting the telephone interview, verbal consent was obtained from each eligible subject willing to participate in the study, using the prepared consent script included in the survey instrument's introduction section. All participants were informed of their right to stop or withdraw from the study at any point during the interview.

#### 2.2.5 Survey administration

The CATI lab team included a manager, an assistant data collection specialist, 6 supervisors, and 42 interviewers. The interviewers worked various 6‐h shifts from 9 a.m. to 9 p.m. daily, except for Fridays, when the workday was from 2 to 9 p.m.

CATI technology is a form of computer‐assisted telephone interviewing wherein interviewers use a computer to dial phone calls and enter the respondent's answers directly into the digital device used for data collection. It ensures accurate implementation of skip logic and randomization (Lavrakas, 2008; Tucker & Lepkowski, 2007). Our team of interviewers used convertible laptops with Cisco Communicator software for making calls and Blaise Data Entry Client software to read the programmed WMHQ survey instrument and enter the information collected into the tablets.

The CATI team administered the WMHQ survey using this technology throughout the pilot and two production waves. The pilot was conducted from October 21, 2020, to December 20, 2020. The first wave commenced on January 20, 2021, and completed on April 12, 2021. The second wave ran from May 25, 2021, until July 15, 2021, and then resumed after the summer break, continuing from September 19, 2021, until January 2022. SESRI does not conduct surveys during the summer, especially those involving Qatari citizens, as many potential respondents travel during these months, leading to potential coverage bias.

In order to obtain a representative sample of respondents, maximum effort was made to reach the target population for the survey. One approach to achieving this was releasing the phone numbers in the sample for interviewing in batches, ensuring that the complete call procedures were followed for all numbers. The procedure for setting up batches within the sample involved calling each case in the batch up to two times before releasing the next batch.

The use of batches also improved the efficiency of contacting the respondents after they had received a Short Message Service (SMS) text informing them of an upcoming call. Normally, SESRI's CATI lab does not send an SMS to respondents prior to calling, as past research indicated that this had little impact on survey response (Dubary, 2013; Holmes et al., 2017). However, for this study, the SMS was used to leverage the benefits of a large advertising campaign that highlighted the study's approval by health authorities and well‐known local sponsors.

Accordingly, the timing of SMS message batches was managed so that an interviewer could reach the recipients on the same day of receiving the SMS. The Qatar University SMS (QUsms) system released texts individually, with the majority (81%) sent in the morning. Interviewers then began calling that batch 1 h later. The average batch size ranged between 500 and 1000 cases, depending on the estimated availability of interviewers for initial contact.

**FIGURE 2:**
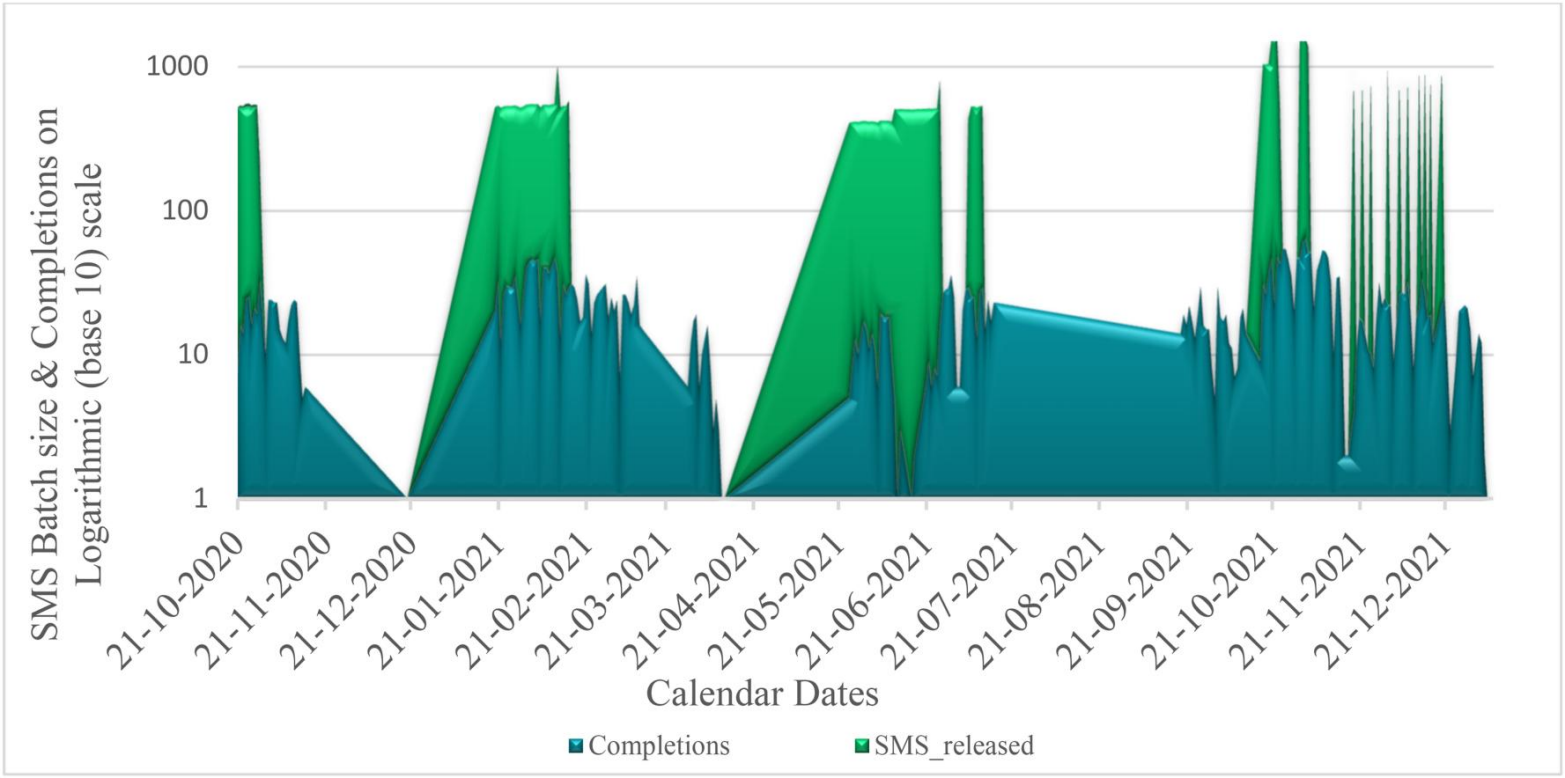
SMS text message release relative to survey completions.

The SMS message identified the sender as associated with Qatar University (“QUsms”). It provided a link to the study's website, which helped explain the purpose of the study. Respondents who wanted to participate but could not find the link were sent a new one upon request. Therefore, in addition to batching SMS messages for subgroups of respondents, our IT team also sent individual SMS requests daily. For Wave 1, 1225 SMS links were sent, and for Wave 2, another 1342 special SMS requests were honored. The pattern of increasing completions relative to the SMS text batches can be observed in Figure 2.

#### 2.2.6 Quality control

The WMHQ's QC system and processes are described in detail elsewhere (Petcu et al., 2023). Here we briefly summarize the main components and procedures that were followed in evaluating the quality of the data.

The team dedicated to QC in the WMHQ survey primarily comprised IT specialists, research assistants, and supervisors from the CATI lab. Monitoring and evaluating the interviewers, in addition to investigating the QC indicators system (QCIS), were key components of the QC process.

##### Quality control system

The QCIS tool was originally developed for the CAPI mode by the SESRI team in collaboration with the Survey Center team at the University of Michigan's Institute for Social Research. It was adapted for survey production in a distributed CATI mode due to changes in the original WMHQ survey methodology necessitated by COVID‐19.

Briefly, the survey data collected by interviewers, along with paradata on their behavior within the survey instrument, were categorized into different sources that fed into the QC system. These sources included the survey entry system, the sample management system, the survey responses, the call‐tracking system, the interviewers' work hours tracking system, and the payroll system. The raw data from these sources were processed at various scheduling frequencies into the QC indicator database.

This database featured an integrated data extraction, transformation, and loading process that further transformed and analyzed the data. The resulting analyses were then displayed on a Power BI dashboard as various indicators and metrics based on different flags created using cut‐offs for data computations. These flags were derived and tested during the phone survey's pilot phase and are referred to by the QC team when monitoring, investigating, and correcting interviewers' performance and behavior in the production phase of the survey.

An intricate flag investigation process was developed (Petcu et al., 2023) to assess the quality of the collected survey data. This process included evaluating the duration of interviews, multiple visits to points within the questionnaire, the speed of reading survey questions, interview pauses, as well as detecting data falsification and improving interviewers' performance and behavior. Two members of the QC team, who reviewed and monitored quality data on a daily basis, conducted these investigations and regularly reported their findings to the research team, CATI lab managers, and supervisors. Details about the QC system including the sources, the processors, the QC indicator dashboard, the developed quality metrics, and flags generated during the WMHQ fielding are described in a recent publication (Petcu et al., 2023).

##### Monitoring

Interviewers who exhibited concerning flags were subjected to further live monitoring by four trained researchers. This live monitoring of interviews allowed us to further investigate suspicious patterns more thoroughly and to understand the reasons behind the triggering of certain flags.

##### Evaluation and interventions

The monitoring team from the research department and CATI Lab supervisors evaluated the interviewers using Power BI and the CATI Time Tracker. The interviewers' skills were rated on a scale from 0 to 5. An award system was implemented to incentivize interviewers who adhered to the interview protocols, triggered a low number of flags, and showed progress after receiving feedback.

Preventive and corrective measures were also taken to reinforce the importance of adhering to the survey interview protocol and guidelines. Preventive measures included monitoring the interviewers' performance for anomalies, reminding them to follow the fieldwork protocols, and providing customized refresher sessions for those needing work on specific weaknesses. To ensure top performance, interviewers were contacted, given feedback, and provided with specific instructions for improvement (up to two times). In cases where there was no improvement, the CATI Lab manager issued a final warning, with dismissal of the interviewer as a last resort.

#### 2.2.7 Weighting procedures

Following data collection, we calculated the weight for each completed response. This calculation included three components: (1) base weights reflecting the sample selection probability; (2) adjustment factors to account for non‐response; and (3) calibration to align the survey results with the population parameters. Additionally, we employed weight trimming, as highly variable weights can introduce undesirable variability in statistical estimates.

First, the base weights were calculated as the inverse of the selection probability of each unit in the sample. Due to the disproportionate sampling described previously in the sample design section, these selection probabilities were necessary to ensure unbiasedness in the analysis: *W*_*base*_ = 1/*p* where *W*_*base*_ is the base weight for the phone number, and *p* is the probability of selection.

Second, the base weights were then adjusted to account for non‐response using this formula: *W* = *αW*_*base*_ where ∝ is the adjustment factor for non‐response. This factor was derived from the propensity of a sampled unit to respond to the survey.

Third, we utilized a calibration weight to align the sample's distributions of sociodemographic characteristics with the known population distributions based on Census data. Calibration helps reduce the effects of bias due to non‐response and under‐coverage of the sampling frame. The “raking” method (Kolenikov, 2014) was employed in the calibration step to adjust the weights of the completed responses. This adjustment ensured that the proportions of the adjusted weights for certain characteristics (such as marital status, gender, and age groups for the Qatari population) aligned with the corresponding proportions in the population.

We also calculated a separate weight variable (W_1_) for those who completed the long interview. Since the selection for the long interview was based on meeting the threshold for mental disorder diagnostic criteria, the weight variable for the long interview was calculated as follows: *W*1 = *β*W, where *β* is the inverse of the proportion of respondents who completed the long interview for each threshold category of mental disorders.

## 3 RESULTS

### 3.1 Interview length

The interview averaged 77 min in length. However, the duration of the interview varied over the course of the survey due to the type of interview (short vs. long) and data collection wave, as shown in Table 3.

**TABLE 3:** Average interview length in minutes by data collection wave, interview type and overall.

Average survey time (minutes)	Pilot	Wave 1	Wave 2	Total sample
Short interview	63.2	58.2	64.5	62.3
Long interview	90.3	84.8	94.0	91.0
Overall (short/long)	78.8	70.9	79.4	76.7

### 3.2 Attempts, completions, & response rates

For every phone number in the sample, there were up to eight attempts to complete the interview. Calls were made at different times during the day and on different days of the week to maximize contact chances with respondents. For phone numbers with break‐offs or soft refusals, dedicated interviewers attempted to recontact and convert these into completed interviews. Supervisors remotely monitored a proportion of calls to ensure QC and adherence to strict protocols for reading the survey instrument. However, due to the sensitive nature of the questionnaire, respondents with two soft refusals were automatically categorized as a hard refusal and removed from further contact. Moreover, in accordance with Qatar's cultural customs, male interviewers did not interview female respondents. If a male respondent was willing to continue the survey without hesitating or otherwise indicating uncertainty, female interviewers proceeded. Otherwise, the case was transferred to the “male only” group of interviewers. The dispositions for all dialed phone numbers during the different phases of the WMHQ survey are shown in Table 4.

**TABLE 4:** Calling dispositions and response rate during different data collection phases of the World Mental Health Qatar survey.

Disposition	Pilot	Wave 1	Wave 2	Total
Completed^a^	447	1551	3197	5195
Not completed	3703	12,499	37,603	53,805
Eligible^b^	910	3685	12,627	17,222
Ineligible^c^	1884	6201	18,274	26,359
Unknown eligibility^d^	909	2613	6702	10,224
Raw response rate RR1,^e^ (%)	19.7	19.8	14.2	15.9
Adjusted response rate or RR2,^f^ (%)	25.7	24.1	16.9	19.2

*Note*: Response rates (RR1 and RR2) were calculated using standardized coding and interpretation procedures for different dialing outcomes, as set by AAPOR (2023).

^a^ People who reached the last question in the survey, namely the demographics section, near the end of the interview (after answering most health‐related questions).

^b^ Eligible phone numbers included Arab residents who refused to participate in the study and those who agreed to an appointment but did not fulfill the appointment upon follow‐up. People who completed part of the interview were also included in this category.

^c^ Ineligible cases primarily consisted of non‐Arab individuals, Arabs who could not speak Arabic, and those under 18 years old.

^d^ Phone numbers dialed with no answer, a hang‐up, or a refusal before interviewers could determine the eligibility of the case.

^e^ Ratio of the number of completed interviews to the total sample size after excluding ineligibles.

^f^ Ratio of the number of completed interviews to the total sample size after accounting for unknown eligibility.

We report two response rates in Table 4. First, the raw response rate, which is the ratio of the number of completed interviews to the total sample size after excluding ineligibles: $\text{RR}1=\frac{C}{C+E+\text{UE}}$ where C is the number of completions, E is the number of eligible responses, and UE is the number of cases with unknown eligibility. Second, the adjusted response rate, $\text{RR}2=\frac{C}{C+E+\text{eUE}}$ where e is the estimated proportion of eligible cases, given by $e=\frac{C+E}{C+E+\text{IE}}$ where IE is the number of ineligible cases. The adjusted rate (RR2) uses a conservative estimate of the proportion of unknown outcome cases that would likely be ineligible.

### 3.3 Percentage of completion by respondent type (Qatari, Arab)

The percentage of completed cases by nationality was similar across the different waves of the study, as shown in Table 5. This distribution approximates the proportion of Arabs (Qatari and non‐Qatari) in Qatar's general population.

**TABLE 5:** Percentage of completion by respondent type (Qatari, non‐Qatari Arab), by data collection wave and overall.

Respondent type	Pilot	Wave 1	Wave 2	Total sample
Qatari (%)	24.5	25.9	29.3	27.8
Arab (%)	75.5	74.1	70.7	72.2
Overall (%)	100.0	100.0	100.0	100.0

The response rate, as shown in Table 6, dropped from an average of 0.38 completions per hour (CPH) at the beginning of wave 1 to only 0.25 in wave 2.

**TABLE 6:** Completions per hour (CPH) and average number of attempts per completion.

Wave	CPH^a^	Average number of dials
Pilot	0.33	3.4
Wave 1	0.38	3.1
Wave 2	0.25	3.9
Overall	0.29	3.6

^a^ CPH: Completed Calls per hour.

## 4 DISCUSSION

We describe above the methodology of the WMHQ survey, Qatar's first national population‐based mental health survey. Conducted as part of the WHO WMH Survey Initiative, this survey took place during the COVID‐19 pandemic. Conducting a long and sensitive telephone survey in a rapidly developing Muslim country amidst a global health crisis presented both traditional and novel challenges. Traditional challenges included adapting an established, lengthy instrument to a mode typically requiring a shorter survey length, dealing with low response rates or non‐response due to the sensitivity of the survey topic, and managing increasing field costs. Novel challenges entailed adapting to a distributed remote CATI system that allowed for quality assurance monitoring, clear voice transmission between interviewer and respondent, centralized remote management, and robust data security. We carefully addressed these challenges and planned accordingly to ensure the quality and accuracy of the survey data.

The trade‐off decisions made by SESRI's WMHQ study group consistently prioritized quality over cost. For example, matching interviewer and respondent gender, especially for female respondents, was necessary due to the conservative norms in a significant portion of the target population. Binding respondents to the same interviewer, even if completed in separate sessions, once a threshold question was reached, helped reassure them about confidentiality. It also made efforts to verify interviewer behavior more clear‐cut. However, this approach required interviewers to be vigilant about appointments and wait for them rather than call new respondents. This process proved more expensive given the average survey length relative to shift duration.

In addition to pilot testing the survey instrument, which identified issues with the survey questions or processes before the main survey, we employed a well‐trained and culturally sensitive survey team. This team no doubt improved response rates and overall data quality and contained a larger than usual number of interviewers who also worked in the health care sector.

We provided clear and concise instructions to participants during the survey, sending them an SMS with a link to the study website. This link explained the study's purpose and offered contact information for any questions or concerns. A few interviewers were tasked with receiving calls from respondents to address or redirect their inquiries. We also monitored social media to respond to negative messaging or misinformation about the study. Finally, we routinely checked phone applications like “True Caller” to see if our outgoing numbers were being reported as spam, issuing new numbers from Qatar University when necessary.

These efforts, combined with the development of in‐house technologies for QC, robust supervision of interviewers, and targeted management of the sample in batches, aided our efforts to increase overall participant satisfaction while reducing survey break‐offs and non‐response. However, conducting a long telephone survey with sensitive questions professionally and ethically is inherently costly. Over time, word of mouth among the public likely contributed to a decrease in response, although we cannot quantify this concern.

Despite proactive efforts, managing interviewer productivity remotely and controlling survey costs remained or even increased as primary challenges. The break‐off rate for the WMHQ survey production was higher than anticipated— 68.0% compared to 49.2% in the phone pilot. This, along with the extended survey field period (from 3 to 4 years), contributed to increased data collection costs. The sample of telephone numbers became older and thus less efficient over time. Furthermore, the interview length increased from a median of 55 min in Wave 1 to 75.8 min in Wave 2. Fewer respondents were answering, and those who did took longer, on average, to complete interviews. Monitoring dissuaded interviewers from trying to shorten their interviews by reading too fast or to “push” respondents to participate. Supervisors were directed to correct any such behavior to maintain data quality and the voluntary nature of participation.

As a result, the study's initial lower estimated costs of interviewing, measured during the phone pilot, were not realized as the average completion per hour (CPH) rate of 0.38 at the beginning of Wave 1 dropped to 0.25 during Wave 2. One strategy to offset an expected loss in efficiency is to mount an effective and robust advertising campaign. However, even with extensive media coverage for the WMHQ throughout the State of Qatar (including national TV, digital billboards in malls, and a social media campaign), the costs of interview completion continued to rise, along with the overall costs of reaching the original target of 5000 interviews. Additionally, the technical costs of measuring interviewer performance increased, and there was less certainty over the per‐interview cost due to the novelty of conducting a lengthy and sensitive survey by phone using a remote or distributed system. For example, it was challenging to determine if long wait times between calls were due to interviewers getting “burned out” and taking longer breaks, or if they were waiting, as instructed, to meet an appointment while being more aware of the potential length of a new interview, which could cause them to miss a promised appointment.

To conduct phone surveys safely amidst a pandemic, we developed several IT tools. These included dashboards for measuring interviewer performance and efficiency, procedures for the direct retrieval of cases, and the programmatic binding of cases to interviewers based on a threshold question. Additionally, SESRI's IT unit developed a smartphone application for tracking interviewers' self‐reported work hours against system time data and another for inputting interviewer monitoring scores and receiving feedback from interviewers. A third application was designed to track complaints received from the public. Furthermore, interviewers communicated with each other, with their supervisors, and with the research team in real time using a chat system.

The IT team also set up a system for the extensive measurement of paradata, which refers to information from a system about a user's behavior on that system. In this case we measured interviewers' movements within the CATI system and their use of the Cisco soft phone on the devices they were issued. For security, these devices were locked down, disabling the USB and other systems. Our paradata captured all keystrokes within the CATI system (including backspace or delete), mouse movements on the screen, time spent on each screen and between screens, time waiting before dialing, the duration of active use of the soft phone, paths through the questionnaire, and many other measures related to their work. Finally, the survey data were analyzed to check for variance in each interviewer's recorded case responses on key questions and demographics compared to those of all other interviewers, aiming to identify systematic differences not explained by chance.

The study required a very large number of respondents in the final dataset to enable statistically high‐powered analyses. To achieve this number of completions while maintaining quality amidst a declining response rate, the research team increased the number of contact attempts per phone number to ensure the sample's representativeness. This reduction in efficiency, combined with the costs of promoting the survey and developing technical means to conduct it in a COVID‐safe manner, led to cost overruns for the WMHQ study. Nevertheless, the study's findings are extremely valuable and innovative, and Qatar's mental health system will benefit from the insights afforded by this dataset for many years to come.

## 5 CONCLUSIONS

In Qatar's inaugural national epidemiological mental health survey, conducted as part of the WHO World Mental Health Survey Initiative, the transition to telephone interviewing during the COVID‐19 pandemic presented a blend of challenges and opportunities. This necessary shift, while complicating budgetary forecasts, resulted in a dataset of high quality, crucial for establishing a baseline for mental disorder epidemiology in a rapidly developing country. The study's findings are poised to aid mental health practitioners and policymakers in the State of Qatar, offering insights for years to come in tracking progress in community‐based mental health services. The methodological adaptations and emerging issues should also be informative to the broader health survey community.

Using a national‐level cellular telephone frame and a distributed network remote CATI system, the WMHQ study navigated many concerns and, in its aftermath, offered practical insights into traditional challenges such as managing interviewer productivity, response rates, costs, and the comparability of measures between different survey modes. In another manuscript for this journal issue, we discuss the survey mode differences in detail (Khaled, Amro, et al., 2024). Notably, the efficiency of the pilot phone study created an appreciable tension between the findings of our pilot mode comparison study and the results presented in this methodological manuscript. In this section, we address some of these tensions.

In short, the findings regarding data quality are very positive, demonstrating the feasibility of telephone interviews as an alternative to face‐to‐face methods within the WMH survey initiative for obtaining comparable mental disorder prevalence estimates. However, costs for the phone survey increased over time. Lessons learned from the pilot may well have contributed to the increased efficiency of Wave 1. This made the inefficiency of Wave 2, which began to rise at the start of the summer before the break and increased even more in the fall, an unpleasant surprise. There is no certain answer as to why this occurred, as some suspected elements cannot easily be quantified. One could summarize the issues as related to sample, word of mouth, the cost of a decentralized CATI system, and easing of COVID‐19 restrictions.

A telephone sample becomes less efficient as it ages. The non‐national population in Qatar, being largely expatriates, is more mobile than in other countries. If a long study duration is anticipated, it might be prudent not to draw a full sample all at once but rather to plan for sample supplements corresponding to study waves. Moreover, in a world where communication on social media and elsewhere has become rapid, it seems likely that word of mouth spread among potential respondents about the length of this national survey. This may have contributed to decreased response rates. Supporting this observation, SESRI had other projects in the field during the WMH study's extended field period and observed similar (albeit less dramatic) increases in nonresponse and survey costs for those studies.

Costs went down for other SESRI phone studies several months after WMH was complete and interviewers had fully returned to using the centralized calling lab. The implementation of a distributed network CATI system, while enabling remote work for interviewers and supervisors, also proved to be more expensive. Monitoring interviewers' productivity and interviewing techniques is more time‐consuming when they are not in a centralized location. This tends to require a higher supervisor‐to‐interviewer ratio if quality is to be maintained, an observation mirrored in other parallel CATI studies at that time. Despite the higher costs, the system was crucial during the pandemic and offered increased efficiencies in specific contexts, such as pretesting, short trainings, or survey instrument review by interviewers. Although Qatar was more conservative than many other countries in easing COVID‐19 restrictions, by the time of the WMHQ Wave 2, far more people were regularly venturing out of their homes. The very conditions that necessitated conducting this survey by phone may have made respondents more willing to engage in a long conversation over the phone during the Pilot and Wave 1 periods. As these conditions diminished, so too did the acceptance of long phone interviews.

The WMHQ study highlights the potential for adapting data collection methodologies in response to unforeseen circumstances, even for extensive and sensitive surveys like those typically conducted within the WMH consortium. It is standard practice in WMH surveys to sometimes complete long interviews requiring multiple follow‐up visits. The results of this study underline the validity of using the phone in this secondary role and suggest the data collected by phone are comparable. The mode effects manuscript in this journal issue further explores this aspect, particularly focusing on the receptiveness of male respondents to phone surveys on this topic (Khaled, Amro, et al., 2024).

Organizations considering a full transition to phone surveys should consider several factors unique to SESRI's context. The public visibility of Qatar University and the trusted reputation of SESRI contribute to achieving higher telephone response rates using a high‐coverage cellular phone sample. SESRI normally has telephone response rates in the 40–60% range for surveys lasting 15–20 min, which is significantly higher than what is typically observed in Western countries for telephone surveys using a probability sample.

Moreover, the high budget initially allocated for a face‐to‐face CAPI study was reallocated to enhance monitoring, technology development, advertising, and respondent outreach for the telephone survey. Despite these advantages, costs rose rapidly halfway through the study, posing concerns for organizations with more limited budgets.

In conclusion, the WMHQ study not only contributes significantly to the understanding of mental health in Qatar but also offers valuable lessons for future mental health surveys globally. Its success in employing a telephone strategy with a long questionnaire on a sensitive topic demonstrates adaptability in research methodologies, particularly in the face of global health emergencies. This study stands as a testament to the feasibility of innovation in methodology to maintain a probability sample where required, as in epidemiological research. It offers a roadmap for future surveys aiming to maintain high standards of data quality and respondent engagement, even in challenging circumstances.

## AUTHOR CONTRIBUTIONS

**Salma M. Khaled**: Conceptualization; funding acquisition; writing ‐ original draft; methodology; writing ‐ review & editing; formal analysis; project administration; data curation; supervision; resources. **Iman Amro**: Writing ‐ original draft; methodology; writing ‐ review & editing. **Lina Bader**: Writing ‐ original draft; writing ‐ review & editing; formal analysis; software. **John Lee Holmes**: Writing ‐ original draft; writing ‐ review & editing; project administration; supervision. **Kien Le Trung**: Writing ‐ review & editing; writing ‐ original draft; Project administration. **Abdoulaye Diop**: Writing ‐ review & editing; supervision; project administration; resources; writing ‐ original draft.

## CONFLICT OF INTEREST STATEMENT

The authors declare no conflicts of interest.

## ETHICS STATEMENT

Qatar University (QU‐IRB 1219‐EA/20) approved the study. The study's goal and methods were explained verbally to participants. Before each survey interview, consent to participate was obtained verbally using a phone script. All data were encrypted and saved on Qatar University's secure server. Each participant was assigned a case number, and individual identifiers were retained in a password‐protected folder only available to the lead principle investigator, senior research assistant, and data analyst. All study researchers, including interviewers, signed confidentiality agreements preventing the sharing or use of participant personal information.

## Data Availability

The data that support the findings of this study are available from Dr. Salma M. Khaled, the principal investigator of the study at skhaled@qu.edu.qa, upon reasonable request and pending additional ethical approval.
